# *In Silico* Characterization of Pectate Lyase Protein Sequences from Different Source Organisms

**DOI:** 10.4061/2010/950230

**Published:** 2010-09-19

**Authors:** Amit Kumar Dubey, Sangeeta Yadav, Manish Kumar, Vinay Kumar Singh, Bijaya Ketan Sarangi, Dinesh Yadav

**Affiliations:** ^1^Department of Biotechnology, D.D.U Gorakhpur University, Gorakhpur 273 009, India; ^2^School of Biotechnology, B.H.U, Varanasi 221 005, India; ^3^Environmental Biotechnology Division, National Environmental Engineering Research Institute, Nehru Marg, Nagpur, Maharashtra 440 020, India

## Abstract

A total of 121 protein sequences of pectate lyases were subjected to homology search, multiple sequence alignment, phylogenetic tree construction, and motif analysis. The phylogenetic tree constructed revealed different clusters based on different source organisms representing bacterial, fungal, plant, and nematode pectate lyases. The multiple accessions of bacterial, fungal, nematode, and plant pectate lyase protein sequences were placed closely revealing a sequence level similarity. The multiple sequence alignment of these pectate lyase protein sequences from different source organisms showed conserved regions at different stretches with maximum homology from amino acid residues 439–467, 715–816, and 829–910 which could be used for designing degenerate primers or probes specific for pectate lyases. The motif analysis revealed a conserved Pec_Lyase_C domain uniformly observed in all pectate lyases irrespective of variable sources suggesting its possible role in structural and enzymatic functions.

## 1. Introduction

The enzymes hydrolyzing pectic substances ubiquitously present in the plant kingdom forming major components of middle lamella are referred as pectinases. The production, purification, biochemical characterization, and application of pectinases have been extensively reviewed [1–10]. The pectinases include polygalacturonases, pectic esterases, pectin lyases, and pectate lyases depending on their mode of action [1]. 

Pectate lyase (PL, EC 4.2.2.2) cleaves the *α*-1,4 glycosidic bonds of polygalacturonic acid via a *β*-elimination reaction producing unsaturated ∆4, 5 bond at the nonreducing end of the polysaccharide and generates 4,5-unsaturated oligogalacturonates. Pectate lyase is widely distributed in diverse families of microorganisms and plants. The important members of bacterial family include *Erwinia carotovora, Bacillus polymyxa, Klebsiella, Yersinia, Cytophaga, Pseudomonas,* and *Xanthomonas* while in fungi *Aspergillus, Fusarium, and Penicillium* are the most predominant source [9, 11–14]. 

A number of pectate lyase genes have been cloned, sequenced, and expressed from different source organism, namely, bacteria [15–22], fungi [23–25], yeast [26], nematode [27] and plants [14, 28].

The three-dimensional structures of various extracellular pectate lyase have been reported [29–36]. The pectate lyases, in general, have a parallel *β*-helix domain formed by parallel-strands folded into a large right-handed helix and a major loop region. 

Amino acid sequence homology-based classification of pectate lyases into distinct families suggesting the possible evolution from different lineages has been reported [20, 35, 37–44]. In *silico* analysis of pectin lyase protein sequences has been recently reported [45].

This paper reports *in silico *characterization of pectate lyase protein sequences from different source organisms for homology search, multiple sequence alignment, phylogenetic tree construction, and motif analysis using various bioinformatics tools.

## 2. Materials and Methods

A total of 121 protein sequences of pectate lyases of different source organism available in GenBank were downloaded from NCBI (http://www.ncbi.nlm.nih.gov/). The accession numbers of pectate lyases protein sequences along with the source organism are listed in Table 1.

The program ClustalW [46] was used for multiple sequence alignment. Mega 4 was used for dendrogram construction by Neighbor-Joining (NJ) method [47]. For domain search, the Pfam site (http://www.sanger.ac.uk/software/pfam/search.html) was used. Domain analysis was done using MEME (http://meme.sdsc.edu/meme/meme.html) [48]. The conserved protein motifs deduced by MEME were characterized for biological function analysis using protein BLAST, and domains were studied with Interproscan providing the best possible match based on highest similarity score.

## 3. Results and Discussion

**Figure 1:**
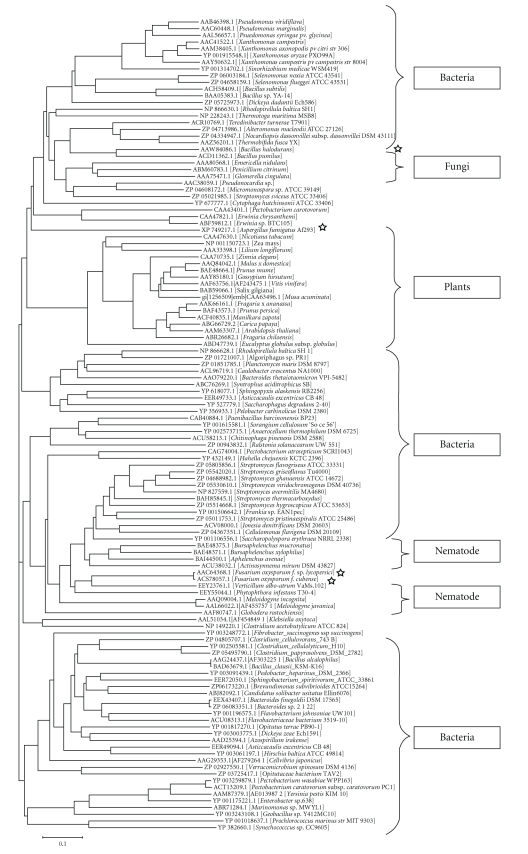
Phylogenetic tree of pectate lyase protein sequences from different source organism constructed by NJ method.

A total of 121 pectate lyases sequences from different source organisms subjected to phylogenetic tree construction revealed major clusters of bacterial, fungal, plant, and nematode pectate lyases. The pectate lyase from bacterial source was the predominant comprising of 87 accession numbers. The different accession of bacterial pectate lyase formed three major clusters as shown in Figure 1. The plant, fungal, and nematode pectate lyases formed separate clusters signifying the sequence-based similarity with reference to different source organisms. The multiple accessions of bacterial, fungal, plant, and nematode pectate lyases were placed closely in the clusters signifying the greater degree of sequence level similarity. Similar phylogenetic tree revealing clustering of pectin lyases protein sequences based on different source organism has been reported [45].

**Figure 2:**
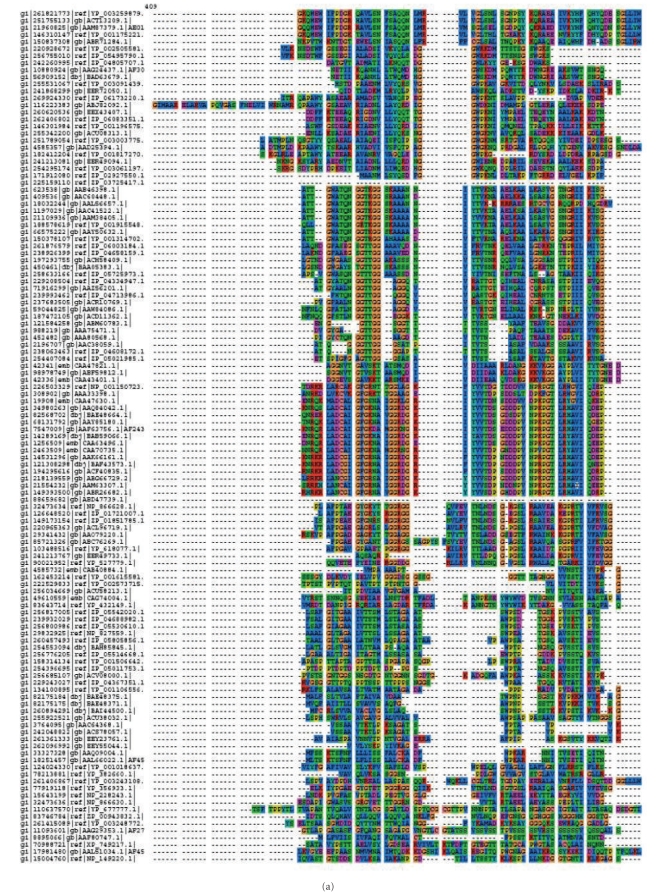

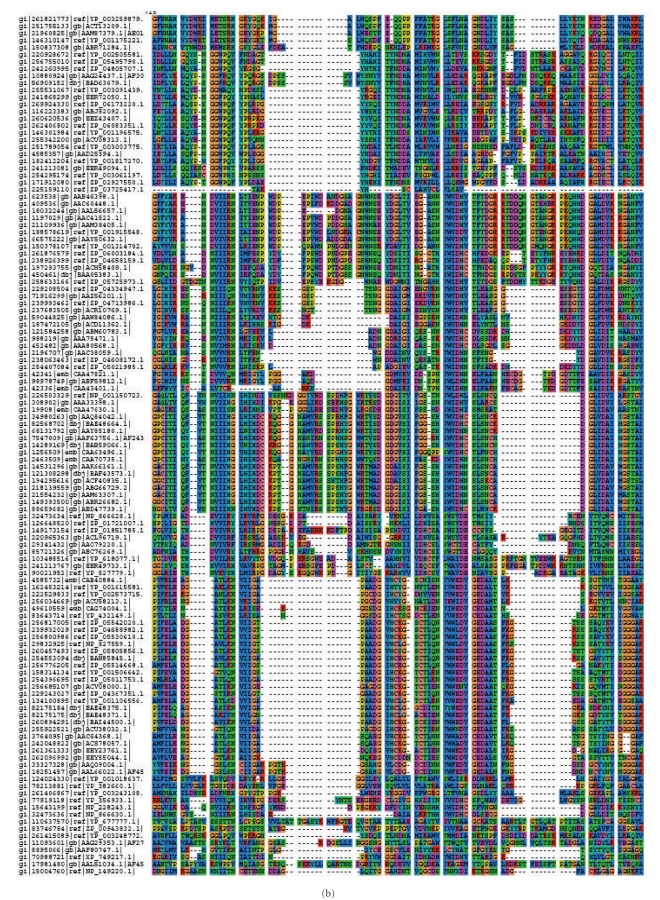

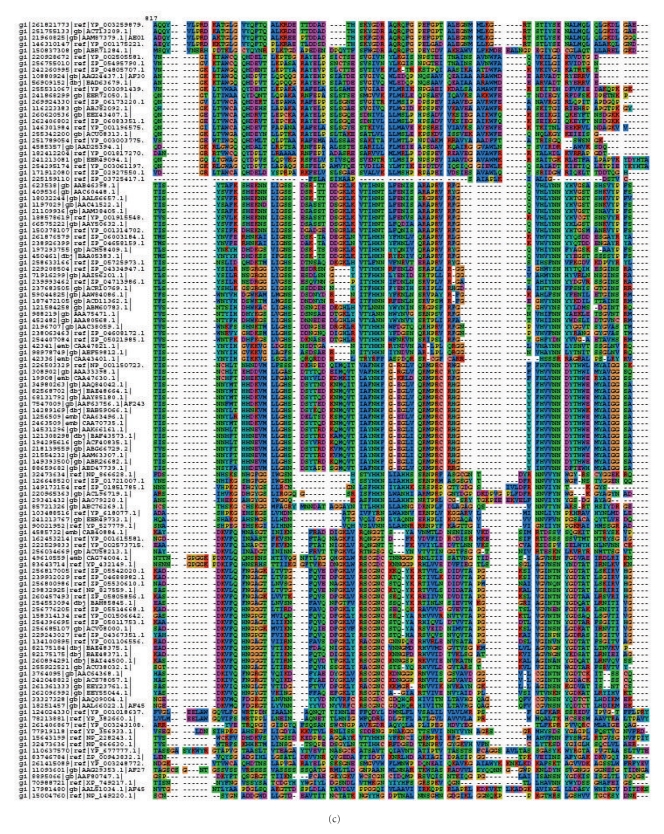
(a) Multiple sequence alignment of pectate lyase protein sequences showing maximum homology from amino acid residues 439–467. (b) Multiple sequence alignment of pectate lyase protein sequences showing maximum homology from amino acid residues 715–816. (c) Multiple sequence alignment of pectate lyase protein sequences showing maximum homology from amino acid residues 829–910.

The multiple sequence alignment of these protein sequences revealed conserved regions at different stretches, namely, from 439–467, 715–816, and 829–918 amino acid residues (Figures 2(a), 2(b), and 2(c)). This region could be used for designing degenerate primers or probes for PCR-based amplification or hybridization-based detection of pectate lyase sequences from different source organisms. 

A total of five motifs labelled as 1, 2, 3, 4, and 5 were observed in only 91 sequences when subjected to MEME. The distribution of these motifs among 92 pectate lyase accession number is shown in Table 2. 

The motifs with width and best possible match amino acid sequences are shown in Table 3. All these motifs showed similarity with pec_lyase_C domain which is quite prevalent in pectin lyase sequences as reported earlier [45]. As the mechanism of pectin lyase and pectate lyase is quite similar, it is expected to show similar motifs. The motif 1 of 29 amino acid residues with sequence IAFNHFGEGLVQRMPRCRHGYFHVVNNDY and motif 4 of 50 amino acid residues with a sequence HNSLSNCHDGLIDAIHGSTAITISNNYMTHHDKVMLLGHSDSYTQDKNMQ were observed in 47 and 39 pectate lyase protein sequences (Table 3) signifying their possible role in the structural and catalytic attributes of pectate lyases. 

Further when the motif best possible match amino acid sequence was subjected to BLAST to reveal its identity, it was observed that the motifs 1, 2, 3, and 4 represents Pec_Lyase_C superfamily while motif 5 represents pectate lyase superfamily. The exact function of these motifs in influencing the catalytic activity of the pectate lyase needs to be investigated. 

 The *in silico* characterization of pectate lyases protein sequences from different source organisms has revealed sequence level similarity specific for different groups which could be utilized for designing strategy for cloning the putative genes based on PCR amplification using degenerate primers.

## Figures and Tables

**Table 1:** List of pectate lyase protein sequences with respective accession number from different source organisms.

Group	Total number	Accession number (Source organism name)
Nematode	06	AAQ09004.1[*Meloidogyne incognita*], AAL66022.1|AF455757 1[*Meloidogyne javanica*], AAF80747.1[*Globodera rostochiensis*], BAE48375.1[*Bursaphelenchus mucronatus*], BAE48371.1[*Bursaphelenchus xylophilus*], BAI44500.1[*Aphelenchus avenae*]

Plant	17	CAA47630.1[Nicotiana tabacum], NP 001150723.1[Zea mays], AAA33398.1[Lilium longiflorum], CAA70735.1[Zinnia elegans], AAQ84042.1[Malus x domestica], BAE48664.1| Prunus mume], AAY85180.1[Gossypium hirsutum], AAF63756.1|AF243475 1[Vitis vinifera], BAB59066.1[Salix gilgiana], gi|1256509|emb|CAA63496.1[Musa acuminata], AAK66161.1[Fragaria x ananassa], BAF43573.1[Prunus persica], ACF40835.1[Manilkara zapota], ABG66729.2[Carica papaya], AAM63307.1[ Arabidopsis thaliana], ABR26682.1[Fragaria chiloensis], ABD47739.1[Eucalyptus globulus subsp. Globules

Fungi	10	AAA80568.1[*Emericella nidulans*], ABM60783.1[*Penicillium citrinum*], AAA75471.1[*Glomerella cingulata*], AAC64368.1[*Fusarium oxysporum f. sp. lycopersici*], ACS78057.1[ *Fusarium oxysporum f. cubense*], EEY55044.1[*Phytophthora infestans* T30-4], XP 749217.1[*Aspergillus fumigatus Af*293], AAA80568.1[*Emericella nidulans*], ABM60783.1[*Penicillium citrinum*], EEY23761.1[*Verticillium albo-atrum* VaMs.102]

Bacteria	87	AAB46398.1[*Pseudomonas viridiflava*]*, AAC60448*.1[*Pseudomonas marginalis*]*, AAL*56657.1[*Pseudomonas syringae pv. glycinea*], AAC41522.1[*Xanthomonas campestris*], AAM38405.1[*Xanthomonas axonopodis pv citri str* 306], YP 001915548.1|[*Xanthomonas oryzae PXO99A*]*, AAY*50632.1|[*Xanthomonas campestris pv campestris str* 8004], YP 001314702.1[*Sinorhizobium medicae WSM419*]*, ZP *06003184.1[*Selenomonas noxia ATCC* 43541], ZP 04658159.1[*Selenomonas flueggei ATCC* 43531], ACH58409.1|[*Bacillus subtilis*], BAA05383.1[*Bacillus sp. YA-14*]*, ZP* 05725973.1[*Dickeya dadantii Ech586*], NP 866630.1[*Rhodopirellula baltica SH1*], NP 228243.1[*Thermotoga maritima MSB8*], ACR10769.1[*Teredinibacter turnerae *T7901], ZP 04713986.1[*Alteromonas macleodii *ATCC 27126], ZP 04334947.1[*Nocardiopsis dassonvillei subsp. dassonvillei DSM* 43111], AAZ56201.1[*Thermobifida fusca YX*], AAW84086.1[*Bacillus halodurans*], ACD11362.1[*Bacillus pumilus*], AAC38059.1[*Pseudonocardia sp*.], ZP 04608172.1[*Micromonospora sp ATCC* 39149], ZP 05021985.1[*Streptomyces sviceus* ATCC 33406], YP 677777.1[*Cytophaga hutchinsonii* ATCC 33406], CAA43401.1[*Pectobacterium carotovorum*], CAA47821.1[*Erwinia chrysanthemi*], ABF59812.1[*Erwinia sp. BTC105*], NP 866628.1[*Rhodopirellula baltica SH 1*], ZP 01721007.1[*Algoriphagus sp PR1*], ZP 01851785.1[*Planctomyces maris DSM *8797], ACL96719.1[*Caulobacter crescentus* NA1000], AAO79220.1[*Bacteroides thetaiotaomicron VPI-*5482], ABC76269.1[*Syntrophus aciditrophicus SB*], YP 618077.1[*Sphingopyxis alaskensis *RB2256], EER49733.1[*Asticcacaulis excentricus *CB 48], YP 527779.1[*Saccharophagus degradans *2-40], YP 356933.1[*Pelobacter carbinolicus DSM 2380*], CAB40884.1[*Paenibacillus barcinonensis BP23*], YP 001615581.1[*Sorangium cellulosum 'So ce* 56'], YP 002573715.1[*Anaerocellum thermophilum DSM *6725], ACU58213.1[*Chitinophaga pinensis DSM 2588*], ZP 00943832.1[*Ralstonia solanacearum UW* 551], CAG74004.1[*Pectobacterium atrosepticum* SCRI1043], YP 432149.1[*Hahella chejuensis KCTC* 2396], ZP 05805856.1[Streptomyces flavogriseus ATCC 33331], ZP 05542020.1[Streptomyces griseoflavus Tu4000], ZP 04688982.1[Streptomyces ghanaensis ATCC 14672], ZP 05530610.1[Streptomyces viridochromogenes DSM 40736], NP 827559.1[Streptomyces avermitilis MA-4680], BAH85845.1| [Streptomyces thermocarboxydus], ZP 05514668.1[Streptomyces hygroscopicus ATCC 53653], YP 001506642.1[Frankia sp EAN1pec], ZP 05011753.1[Streptomyces pristinaespiralis ATCC 25486], ACV08000.1[Jonesia denitrificans DSM 20603], ZP 04367351.1[Cellulomonas flavigena DSM 20109] YP 001106556.1[*Saccharopolyspora erythraea* NRRL 2338], ACU38032.1[*Actinosynnema mirum* DSM 43827], AAL51034.1|AF454849 1[*Klebsiella oxytoca*], NP 149220.1[*Clostridium acetobutylicum* ATCC 824], YP 003248772.1[*Fibrobacter_succinogenes ssp succinogens*], ZP 04805707.1[*Clostridium_cellulovorans_*743B], YP002505581.1[*Clostridium_cellulolyticum_*H10], ZP 05495790.1[*Clostridium_papyrosolvens_DSM*_2782], AAG24437.1|AF303225 1[*Bacillus alcalophilus*], BAD63679*.1*[*Bacillus_clausii_KSM-K16*], YP 003091439.1[*Pedobacter_heparinus_*DSM_2366], EER72050.1[*Sphingobacterium_spiritivorum*_ATCC_33861], ZP 06173220.1[*Brevundimonas subvibrioides* ATCC15264], ABJ82092.1[*Candidatus solibacter usitatus Ellin*6076], EEX43407.1[*Bacteroides finegoldii* DSM 17565], ZP 06083351.1[*Bacteroides sp.* 2 1 22], YP 001196575.1[*Flavobacterium johnsoniae* UW101], ACU08313.1[*Flavobacteriaceae bacterium* 3519-10], YP 001817270.1[*Opitutus terrae* PB90-1], YP 003003775.1[*Dickeya zeae* Ech1591], AAD25394.1[*Azospirillum irakense*], EER49094.1[*Asticcacaulis excentricu*s CB 48], YP 003061197.1[*Hirschia baltica* ATCC 49814], AAG29353.1|AF279264 1[*Cellvibrio japonicus*], ZP 02927550.1[*Verrucomicrobium spinosum *DSM 4136], ZP 03725417.1[*Opitutaceae bacterium* TAV2], YP 003259879.1[*Pectobacterium wasabiae* WPP163], ACT13209.1[*Pectobacterium carotovorum subsp. carotovorum* PC1], AAM87379.1|AE013987 2[*Yersinia pestis KIM* 10], YP 001175221.1[*Enterobacter *sp.638], ABR71284.1[*Marinomonas* sp.MWYL1], YP 003243108.1[*Geobacillus sp.* Y412MC10], YP 001018637.1[*prochlorococcus marinus* str MIT 9303], YP 382660.1[*Synechococcus sp. *CC9605]

**Table 2:** Distribution of motifs among 91 pectate lyase proteins sequences from different source organisms.

S. no.	Accession no.	Motif 1	Motif 2	Motif 3	Motif 4	Motif 5
1	AAB46398	+			+	
2	CAB40884					+
3	CAA47630	+	+	+	+	
4	CAA70735	+	+	+	+	
5	CAA43401				+	
6	CAA47821	+				
7	NP866630	+	+		+	
8	YP003003775				+	
9	ZP05725973	+			+	
10	NP866628	+	+		+	+
11	ZP05805856					+
12	YP002573715					+
13	YP001506642					+
14	NP001150723	+	+	+	+	
15	BAE48375					+
16	BAE48371					+
17	AAD25394	+				
18	ACV08000					+
19	ACU58213					++
20	AAC60448	+	+		+	
21	BAI44500					+
22	YP001314702	+			+	
23	AAQ09004					+
24	AAL66022.1|AF455757_1					+
25	AAF80747					+
26	AAC64368					+
27	AAA33398	+	+	+	+	
28	ACU38032					+
29	ACR10769	+	+	+		
30	ACU08313				+	
31	BAH85845					+
32	ACS78057					+
33	ZP04367351					+
34	AAW84086	+		+		
35	AAL56657	+	+	+	+	
36	AAK66161	+	+	+	+	
37	AAQ84042	+	+	+	+	
38	AAC41522	+			+	
39	AAA80568	+			+	+
40	ZP06003184	+			+	
41	ACH58409	+		+	+	
42	AAF63756.1|AF243475_1	+	+	+	+	
43	ZP04713986	+				
44	ZP04334947	+		+		
45	ABG66729	+	+	+	+	
46	ABR26682	+	+	+	+	
47	ACD11362	+		+		
48	XP749217	+			+	
49	BAA05383	+			+	
50	BAB59066	+	+	+	+	
51	ZP01851785	+	+			+
52	ABM60783	+			+	+
53	BAF43573	+	+	+	+	
54	ABF59812	+				
55	AAY85180	+	+	+	+	
56	EEY55044					+
57	EEY23761				+	+
58	YP001615581				+	+
59	ZP05542020					+
60	ZP05530610					+
61	ZP05514668					+
62	ZP05011753					+
63	ZP04688982					+
64	YP001106556					+
65	NP827559					+
66	YP432149					+
67	ABD47739	+		+	+	
68	ZP00943832					+
69	AAM63307	+	+	+	+	
70	AAC38059	+	+		+	
71	AAA75471	+			+	
72	ACL96719	+	+			
73	AAZ56201	+		+		
74	AAY50632	+			+	+
75	AAM38405	+			+	
76	YP618077	+	+			
77	ZP04608172	+			+	
78	ZP05021985	+			+	
79	ZP04658159	+			+	
80	YP001915548	+			+	
81	NP228243	+	+		+	
82	YP356933		+			
83	YP677777	+			+	
84	ACF40835	+	+	+	+	
85	CAA63496	+	+	+	+	
86	AAO79220	+	+	+		
87	ABC76269	+	+			
88	ZP01721007	+	+			
89	BAE48664	+	+	+	+	
90	EER49733	+	+			
91	AAL51034.1|AF454849_1				+	
92	YP527779.1|		+			

**Table 3:** Different motifs commonly observed in pectate lyases protein sequences with best possible match amino acid sequences.

Motif number	Width	Sequence	Occurrence in pectate lyase protein sequences
1	29	IAFNHFGEGLVQRMPRCRHGYFHVVNNDY	47
2	50	NPRPGTLRHAVIQDEPLWIVFKRDMVIQLKQELIMNSFKTIDGRGVNVHI	16
3	50	CITIQFVTNIIIHGIHIHDCKPTGNAMVRSSPSHYGWRTMADGDGISIFG	16
4	50	HNSLSNCHDGLIDAIHGSTAITISNNYMTHHDKVMLLGHSDSYTQDKNMQ	39
5	49	SSSQTMTVDGGGARYAHDKVFQHNGPGTFVIKNFQVQDFGKLYRSCGNC	27
